# Supplementary material for: A New Artificial Intelligence-Based Method for Identifying Mycobacterium Tuberculosis in Ziehl–Neelsen Stain on Tissue
Source: Diagnostics (Basel). 2022 Jun 17;12(6):1484. doi: 10.3390/diagnostics12061484 (PMC9221616; doi:10.3390/diagnostics12061484)
Supplement: Supplementary file 1 [file diagnostics-12-01484-s001.zip › diagnostics-1756715-supplementary.pdf]

diagnostics

MDPI

Table S1. Processing protocol for automatic tissue processor Leica ASP 200S.

No.	Reagent	Time	Temperature	Pressure/ Vacuum	Time of Draining
**1.**	water	10 minutes	-	P/V	80 sec.
**2.**	Ethanol 70°	1½ h	40°C	P/V	80 sec.
**3.**	Ethanol 80°	1¾ h	40°C	P/V	80 sec.
**4.**	Ethanol 96°	1¾ h	40°C	P/V	80 sec.
**5.**	Ethanol 100°	1 h	40°C	P	80 sec.
**6.**	Ethanol 100°	1½ h	40°C	P/V	80 sec.
**7.**	Ethanol 100°	1½ h	40°C	P/V	80 sec.
**8.**	Xylene	2 h	52°C	P	80 sec.
**9.**	Xylene	2 h	52°C	P	80 sec.
**10.**	Xylene	2 h	55°C	P/V	80 sec.
**11.**	Paraffin	1 h	58°C	P	80 sec.
**12.**	Paraffin	2 h	58°C	P	80 sec.
**13.**	Paraffin	3 h	58°C	P	80 sec.

Table S2. Processing protocol for automatic tissue processor Leica Peloris 2.

No.	Reagent	Time	Temperature	Pressure/ Vacuum	Time of Draining
**1.**	Formalin 10%	44 min	45°C	P	10 sec
**2.**	Ethanol100°	30 min	45°C	P	10 sec
**3.**	Ethanol 100°	60 min	45°C	P	10 sec
**4.**	Ethanol 100°	60 min	45°C	P	10 sec
**5.**	Ethanol 100°	60 min	45°C	P	10 sec
**6.**	Ethanol 100°	90 min	45°C	P	10 sec
**7.**	Ethanol 100°	90 min	45°C	P	10 sec
**8.**	Xylene	75 min	45°C	P	10 sec
**9.**	Xylene	75 min	45°C	P	10 sec
**10.**	Xylene	210 min	45°C	P	10 sec
**11.**	Paraffin	120 min	60°C	V	10 sec
**12.**	Paraffin	180 min	60°C	V	10 sec
**13.**	Paraffin	180 min	60°C	V	10 sec

Table S3. Protocol for Ziehl Nielsen stain.

1. Bring section to distilled water.
2. Carbolfuchsin solution - 10 minute; heat with open flame beneath the slide until vapor emission
3. Wash in tap water.
4. Decolorizing solution (Acid differentiation buffer) – 1-2 seconds
5. Wash in tap water.
6. Methylene blue solution 1% – 30 seconds
7. Wash in tap water.
8. Dehydrate through alcohol, xylene and mount

Table S4. Experience of pathologists performing annotations.

Pathologist	Grade	Years of Expertise as Certified Pathologist
**SZ**	Senior pathologist, PhD, professor of pathology	23
**CP**	Senior pathologist, PhD	12
**LN**	Senior pathologist, PhD, lecturer	14
**MiC**	Senior pathologist, PhD student	7
**LS**	Senior pathologist, PhD student	9

**AC**	Pathologist, PhD student	4
**MB**	Pathologist	4

Table S5. Componence of teams of pathologists involved in testing process.

Team of Pathologists	Years of Experience
LN, CP	11-15
LS, MC	6-10
AC, MB	1-5
OS, IT	residents

Table S6. Performance metrics used to evaluate our proposed method.

Performance Metrics	Formula	Equivalent
Sensitivity	TP / (FN + TP)	
Specificity	TN / (FP + TN)	
Precision	TP / TP + FP)	
Negative predictive value	TN / (FN + TN)	
False negative rate	FN / (TP + FN)	1 – sensitivity
False positive rate	FP / (FP + TN)	1 – specificity
Accuracy	(TP + TN) / (TP + TN + FP + FN)	
F1	2TP/(2TP + FP + FN)	

TP – true positive, TN – true negative, FP – false positive, FN – false negative.

Table S7. Errors in WSIs evaluation for qualified pathologists.

**Qualified Pathologists (6 Persons x 60 WSI)**				
		Negative cases	Positive cases	total
**No of errors per WSI**	0	29	10	39
	1	4	6	10
	2	1	6	7
	3	2	0	2
	4	1	1	2
	5	0	0	0
	6	0	0	0
**cases with errors (of 60 WSIs)**		8	13	21
**%**		21.62%	56.52%	35.00%
**No of errors (of 360 examinations)**		16	22	38
**%**		7.21%	15.94%	10.56%

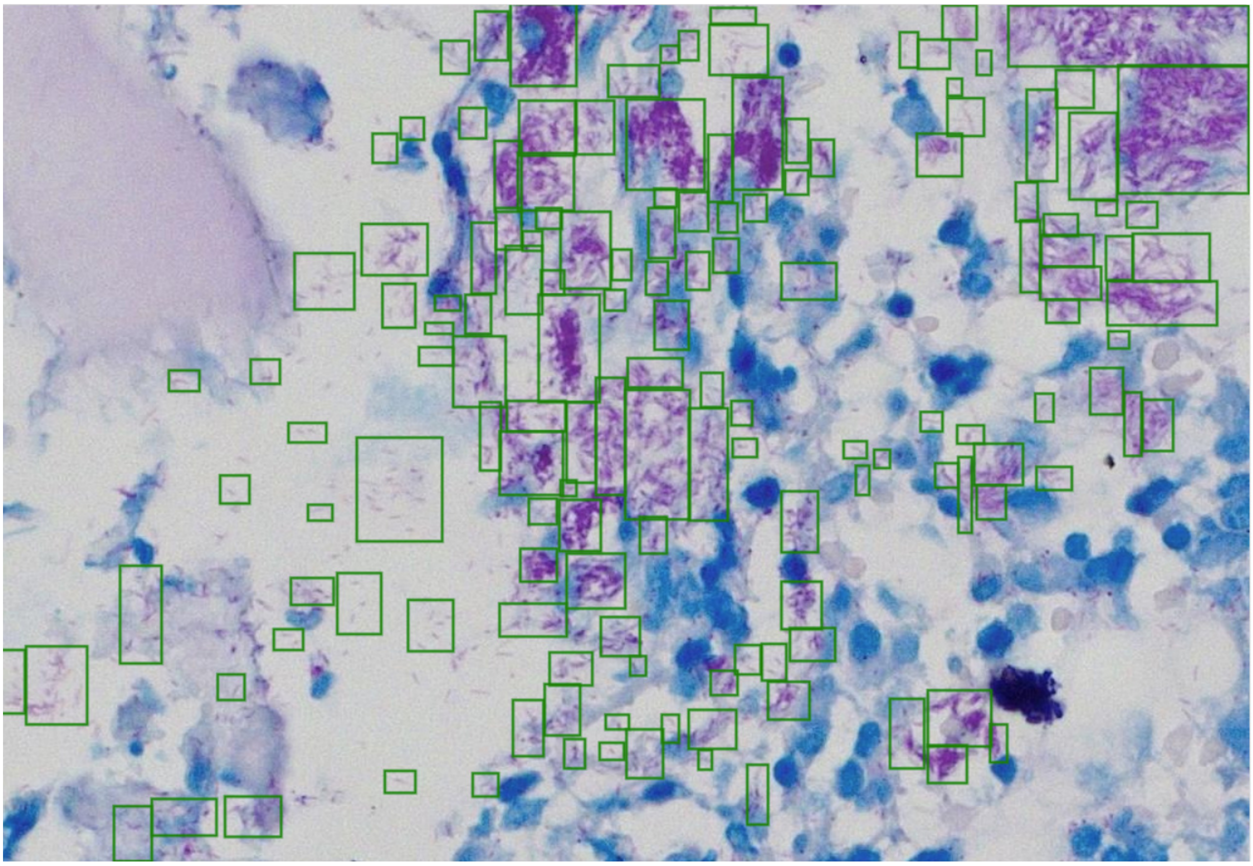

Figure S1. Annotation of AFB in in-house platform with green boxes encircling small groups of bacilli. ZN x 400.

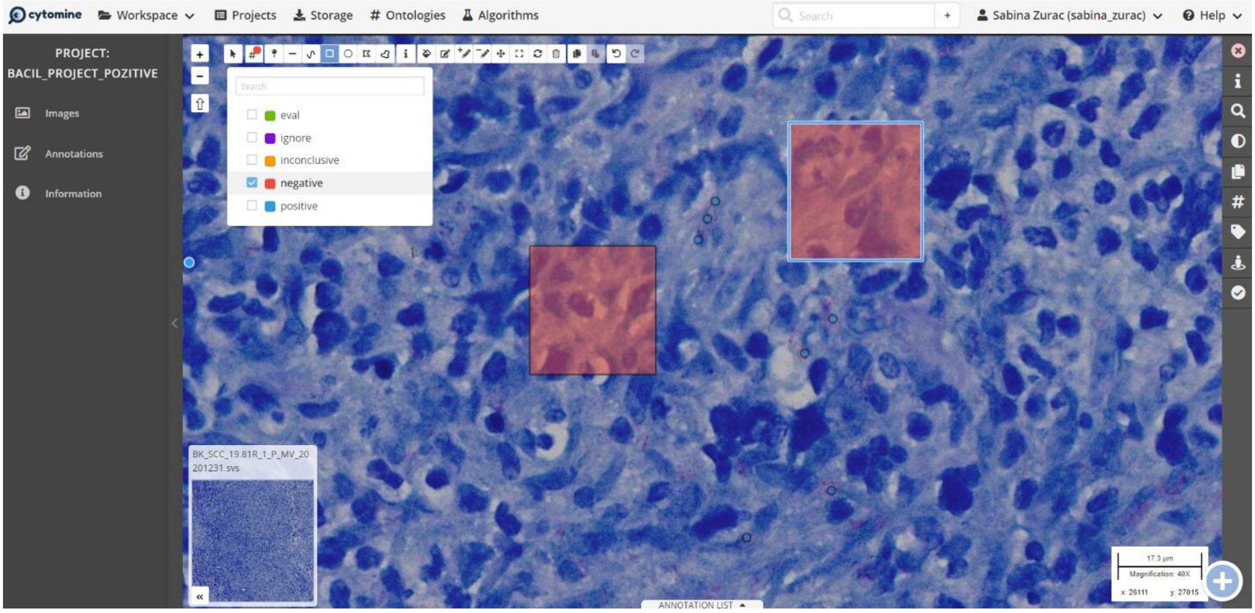

Figure S2. Annotation of AFB in Cytomine platform with blue dots on top of the bacilli and negative red square areas. Tuberculous lymphadenitis with very few bacilli. ZN x 400.

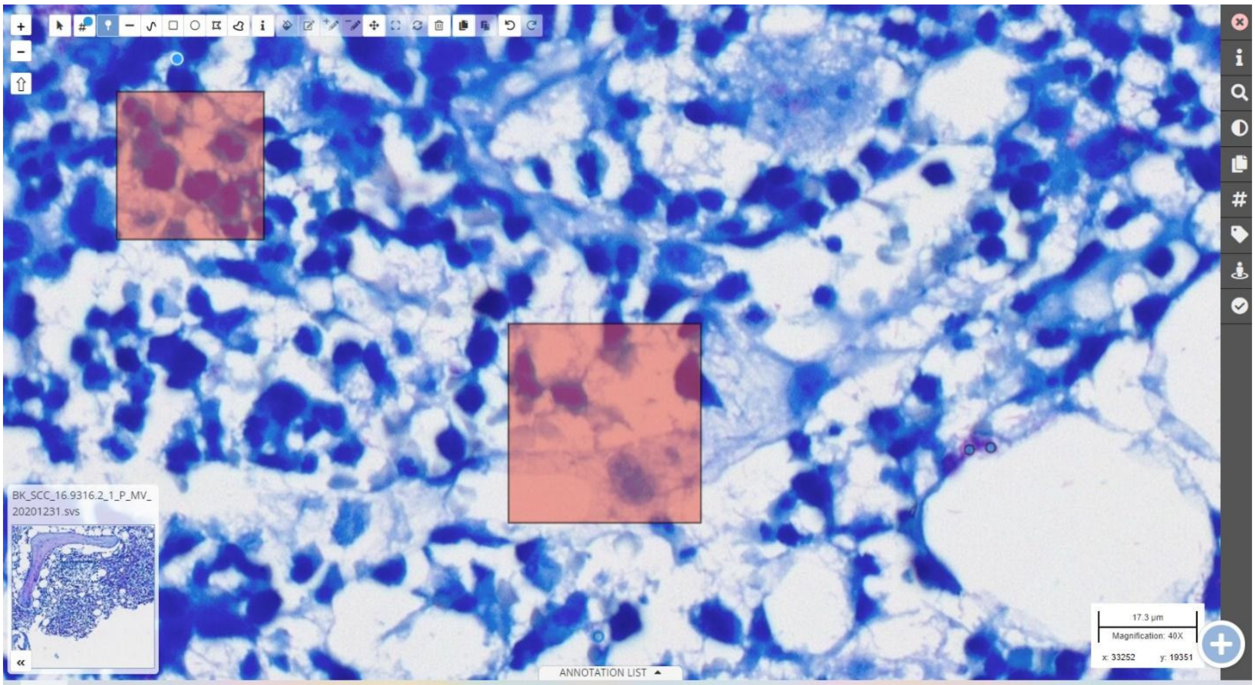

Figure S3. Annotation of AFB in Cytomine platform with blue dots on top of the bacilli and negative red square areas. Bone marrow with variable distribution of AFB. ZN x 400.

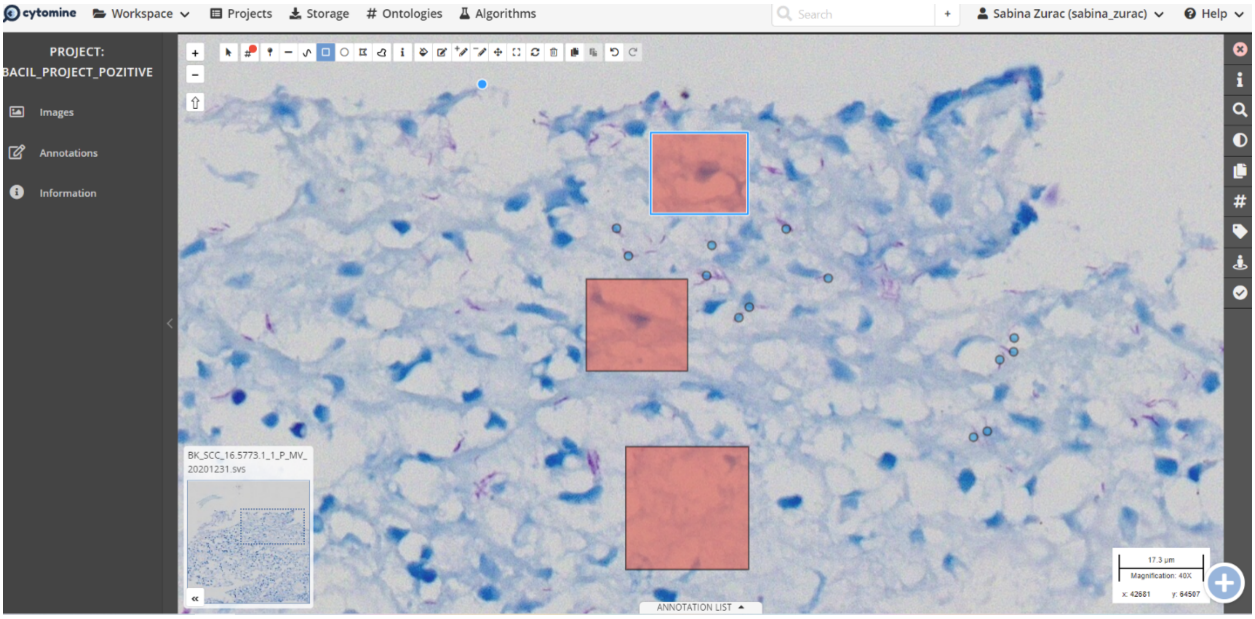

Figure S4. Annotation of AFB in Cytomine platform with blue dots on top of the bacilli and negative red square areas. Case with numerous AFB. ZN x 400.

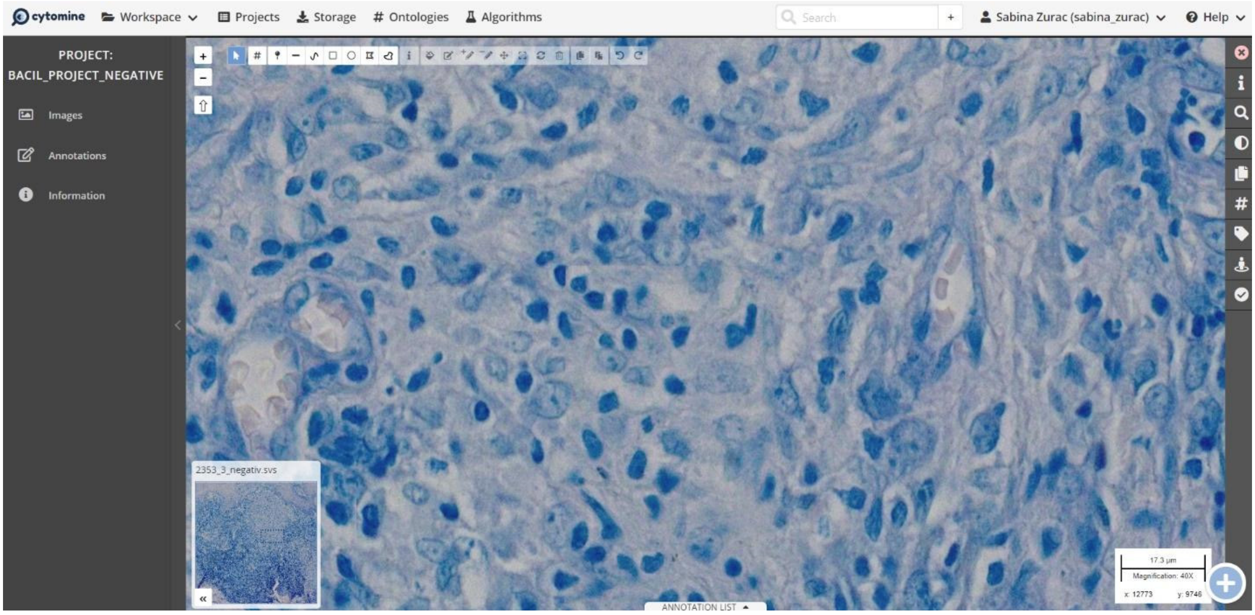

Figure S5. Negative case – granulation tissue. ZN x 400.

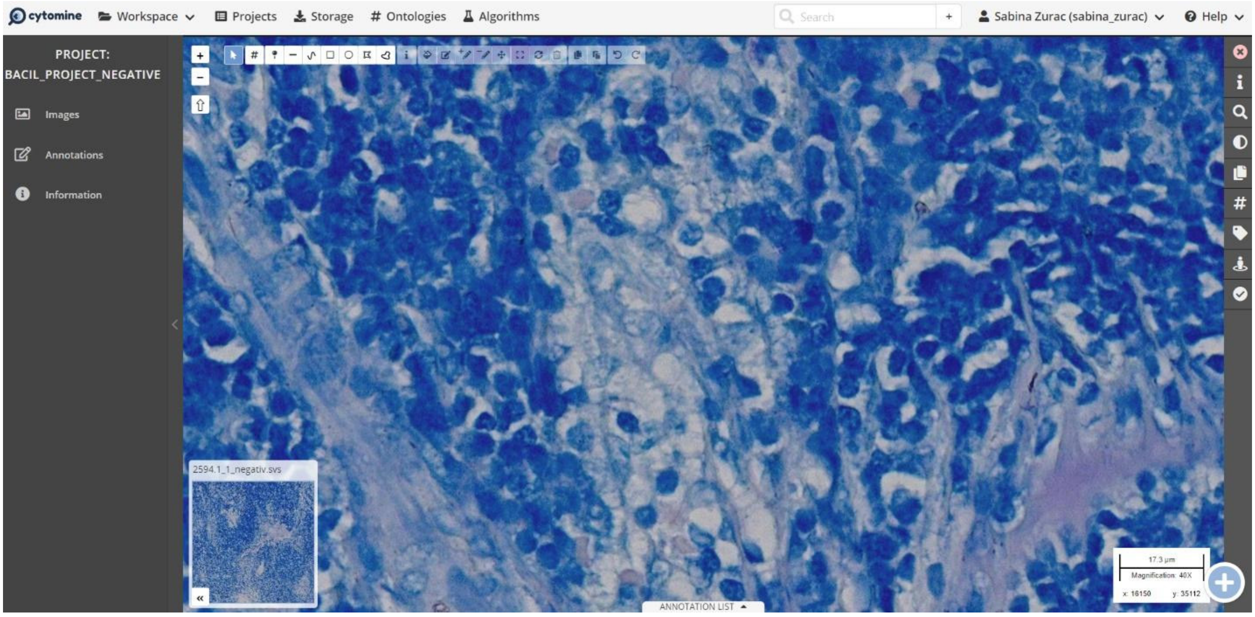

Figure S6. Negative case – reactive lymphadenitis with sinusal histiocytosis. ZN x 400.
